# Extraction Optimization of *Tinospora cordifolia* and Assessment of the Anticancer Activity of Its Alkaloid Palmatine

**DOI:** 10.1155/2013/376216

**Published:** 2013-11-28

**Authors:** Huma Ali, Savita Dixit

**Affiliations:** Department of Chemistry, MANIT, Bhopal, Madhya Pardesh, India

## Abstract

*Objective*. To optimize the conditions for the extraction of alkaloid palmatine from *Tinospora cordifolia* by using response surface methodology (RSM) and study its anticancerous property against 7,12-dimethylbenz(a)anthracene (DMBA) induced skin carcinogenesis in Swiss albino mice. *Methods*. The effect of three independent variables, namely, extraction temperature, time, and cycles was investigated by using central composite design. A single topical application of DMBA (100 **μ**g/100 **μ**L of acetone), followed 2 weeks later by repeated application of croton oil (1% in acetone three times a week) for 16 weeks, exhibited 100 percent tumor incidence (Group 2). *Results*. The highest yield of alkaloid from *Tinospora cordifolia* could be achieved at 16 hours of extraction time under 40°C with 4 extraction cycles. Alkaloid administration significantly decreases tumor size, number, and the activity of serum enzyme when compared with the control (Group 2). In addition, depleted levels of reduced glutathione (GSH), superoxide dismutase (SOD), and catalase and increased DNA damage were restored in palmatine treated groups. *Conclusion*. The data of the present study clearly indicate the anticancer potential of palmatine alkaloid in DMBA induced skin cancer model in mice.

## 1. Introduction

*Tinospora cordifolia* commonly known as Guduchi or Amrita (Menispermaceae), a traditional herbal medicine, is used as a remedy for fever, diabetes, dyspepsia, jaundice, and skin diseases [1]. It has been subjected to extensive phytochemical, pharmacological, and clinical investigation with many interesting findings in the area of immunomodulation, anticancer, hypoglycemic, antiallergic, and anti-inflammatory [2]. Naturally occurring phytochemicals display an active cancer preventive strategy to inhibit, delay, or reverse human carcinogenesis. Studies have indicated that certain daily consumed dietary phytochemicals have cancer protective effects mediated by carcinogens.

Palmatine is a quaternary protoberberine alkaloid. It is typically yellow in color and reported as the most important pharmacological active constituents of a number of plants, such as *Tinospora cordifolia* [3]. Palmatine is a close structural analog of berberine that has been shown to exhibit significant antitumor activity against HL-60 leukemic cells [4]. The alkaloid has been used in the treatment of jaundice, dysentery, hypertension, inflammation, and liver-related diseases. It has also been reported that some of the analogues of palmatine possess antimicrobial and antimalarial activities [5]. The anticarcinogenic activity of palmatine has not yet been fully explored. Cancer prevention could be achieved by avoidance of cancer causing substances and by using chemopreventive agents that can inhibit initiation and also to act as blocking and suppressing agents [6]. It is also an antioxidant and therefore plays a role in preventing some cancers [7].

The purpose of the present study was to optimize the extraction conditions for the isolation of palmatine from *Tinospora cordifolia* on the basis of central composite design and to understand the chemopreventive potential of palmatine in DMBA and croton oil induced skin carcinogenesis mouse model system.

## 2. Material and Methods

### 2.1. Plant Materials

The stems of *Tinospora cordifolia *were collected from MANIT Campus, Bhopal, M.P., India. The plant material was taxonomically identified by Dr. Zia-UL-Hassan, Department of Botany, Saifia College of Science, Bhopal. The voucher specimen (351/Bot/Saifia/12) was preserved in the above herbarium for future reference.

### 2.2. Extraction and Yield of Tinospora Cordifolia

Stems of *Tinospora cordifolia *were dried under shade for 7–10 days and pulverized using an electric grinder. Firstly, dried sample was extracted with solvent of methanol and acetone in the ratio of 70 : 30 (4000 mL × 4 cycles) at 40°C for 16 hours in soxhlet apparatus. The residue was dried under reduced pressure by using a rotary vacuum evaporator.

### 2.3. Experimental Design for Extraction

Response surface analysis was performed to estimate the effects of independent variables on the response within the range of investigation. RSM with the central composite design was used to analyze the experimental data with 3 independent variables (*X*_1_, extraction temperature (°C); *X*_2_, extraction time (hours); *X*_3_ extraction cycles (cycle)) at 5 levels in the extraction process. Investigated factors and tested levels are reported (Table 1).

Table 2 presented the range of independent variables, their ranges and the whole design consisted of 19 experimental points carried out in a random order to optimize the extraction process. Experimental data were added to a second-order polynomial model and regression coefficients were determined. The generalized second-order polynomial model used in the RSM was as follows

(1) $$\begin{array}{c} Y=\beta_{0}+\overset{3}{\underset{i=1}{\sum }}\beta_{i}X_{i}+\overset{3}{\underset{i=1}{\sum }}\beta_{ii}X_{i}^{2}+\overset{3}{\underset{i<j=1}{\sum }}\beta_{ij}X_{i}X_{j}, \end{array}$$

where, *β*_0_, *β*_*i*_, *β*_*ii*_, and *β*_*ij*_ are the regression coefficients for intercept, linear, quadratic, and interaction terms, respectively. *Y* is the yield of *Tinospora cordifolia *and, *X*_*i*_ and *X*_*j*_ are the independent variables.

### 2.4. Isolation of Palmatine from Tinospora Cordifolia

Methanolic extract of stem was partitioned to CHCl_3_ and aqueous extract. The CHCl_3_ solution was dried and evaporated up to a brownish viscous residue (8 gm). The residue was placed on a silica gel column and eluted with CHCl_3_ and gradually enriched with methanol to afford 5 fractions. Fraction 3 eluted with CHCl_3_-MeOH (10 : 1) was repeated and subjected to silica gel column chromatography to give single compound (2.8 gm). Isolated compound was subjected to ultra-violet, infrared, gas chromatography mass spectrometry, and nuclear magnetic resonance spectroscopy. Spectroscopic analysis revealed the presence of a high content of the alkaloid palmatine. This material was further purified by recrystallization with methanol to yield palmatine (99% purity) [8].

### 2.5. Anticancer Activity

#### 2.5.1. Chemicals

7,12-Dimethylbenz(a)anthracene (DMBA), croton oil, NADH, glutathione reduced (GSH), 5,5-dithiobis-(2-nitrobenzoic acid) (DTNB), and 2-thiobarbituric acid (TBA) were obtained from Sigma-Aldrich, USA. All other chemicals were commercially available and analytical grade.

#### 2.5.2. Animals

Swiss albino mice were selected at random from animal house of the Pinnacle Biomedical Research Institute (PBRI), Bhopal. Animals were housed in polypropylene cages with sterile husk and provided standard pellet (Golden feeds, New Delhi) and water *ad libitum* as their feed throughout the experiment. The animals were maintained with a 12 hour light/dark cycle at 22 ± 2°C at controlled condition. All animal experiments were performed with prior permission of the Institutional Animal Ethics Committee (IAEC) of PBRI, Bhopal (registiration number 1283/c/09/CPCSEA).

#### 2.5.3. Determination of Acute Drug Toxicity

According to Organisation for Economic Co-operation and Development (OECD) 423 guideline, the acute toxicity was performed on mice using different doses of palmatine (100, 200, 400, 600, 800, and 1000 mg/kg) orally on five mice. The mice were observed continuously for the first 2 hours and then occasionally for 24 hours and daily thereafter for 30 days for any signs of morbidity, mortality, peripheral blood changes, and behavioral toxicity. No mice were found to be dead during toxicity studies. So, we have selected the 1/5th and 1/10th of maximum exposed dose.

#### 2.5.4. Experimental Design

Five groups (five animals per group) of Swiss albino mice of either sex were used for the study. Animals were dorsally shaved with hair clipper.

*Group 1.* Mice of this group were given Mili-Q water (10 mL/kg body weight), a normal diet, and tap water ad libitum daily. After 16 weeks, mice were autopsied and the skin of dorsal area was taken for the histopathological studies and blood for biochemical analysis. 

*Group 2*. Group 2 animals were treated with a single dose of DMBA (100 *μ*g/100 *μ*L of acetone) over the shaven area of the skin of the mice; afterwards 1% croton oil was applied to skin 3 times a week up to 16 weeks. At the end of experiment, this was considered as carcinogen control group. 

*Group 3.* Group 3 animals were treated topically and orally (200 mg/kg body weight) with palmatine alkaloids for 16 consecutive weeks and were used to study for DNA damage and biochemical and histopathological alterations induced by palmatine, alone treatment.

*Group 4*. After the single dose of DMBA, Group 4 animals were treated with palmatine (100 mg/kg) orally each day till completion of the experiment. After 14 days of DMBA application, 1% croton oil was applied on skin after one hour of palmatine administration three times a week. 

*Group 5. *After the single dose of DMBA, Group 5 animals were treated with palmatine (200 mg/kg) orally each day till completion of the experiment. After 14 days of DMBA application, 1% croton oil was applied on skin after one hour of palmatine administration three times a week.

Mice were observed each week for incidence of skin tumors and their sizes, body weight and average latency period till 16 weeks [9].

#### 2.5.5. Determination of the Effect of Palmatine on Enzymes Involved in Oxidative Stress

At the end of the experiment, animals of all the groups were sacrificed by cervical dislocation. The animals were immediately dissected to remove their skins which were washed in ice-cold saline (0.9% NaCl) and the extraneous material was removed. It was then weighed and blotted dry. The skin tissue homogenate was prepared in 0.15 M Tris-KCl (pH 7.4) and centrifuged at 12000 rpm for 15 minutes.

For biochemical estimation, postmitochondrial supernatant was used on the same day that animals were killed. The level of lipid peroxidase (LPO), glutathione (GSH), superoxide dismutase (SOD), and catalases was estimated by using different methods [10–13].

#### 2.5.6. Determination of the Effect of Palmatine on Serum Enzyme Analysis

The blood was obtained from all animals by puncturing retro-orbital plexus. At room temperature, the blood samples were allowed to clot for 45 min. Serum was separated by centrifugation at 2500 rpm at 30°C for 15 min and utilized for the determination of various biochemical parameters. The levels of serum glutamate oxalate transaminase (SGOT), serum glutamate pyruvate transaminase (SGPT), alkaline phosphatase (ALP), and serum bilirubin were estimated by using the span diagnostic kit.

#### 2.5.7. Determination of DNA Strand Breakage

Alkaline single cell gel electrophoresis (SCGE) was performed as a three-layer procedure [14] with slight modification [15]. The lymphocytes were separated from blood using histopaque density gradient centrifugation and the cells were diluted 20-fold for the comet assay. Viability of the lymphocyte cells was evaluated by the trypan blue exclusion test method [16]. The tissue sample showing cell viability higher than 84% was further processed for comet assay. In brief, about 15 *μ*L of cell suspension (approximately 20,000 cells) was mixed with 85 *μ*L of 0.5% low melting-point agarose and layered on one end of a frosted glass slide, coated with a layer of 200 *μ*L of 1% normal agarose. It was covered with a third layer of 100 *μ*L low melting-point agarose. After solidification of the gel, the slides were immersed in lysing solution (2.5 MNaCl, 100 mMNa_2_ EDTA, 10 mMTris, pH 10 with 10% DMSO, and 1% Triton X-100 added fresh) overnight at 4°C. The slides were then placed in a horizontal gel electrophoresis unit, immersed in fresh cold alkaline electrophoresis buffer (300 mM NaOH, 1 mM Na_2_ EDTA, and 0.2% DMSO, pH > 13.5), and left in solution for 20 min at 4°C for the DNA unwinding and conversion of alkali-labile sites to single strand breaks. Electrophoresis was carried out using the same solution at 4°C for 20 min, using 15 V (0.8 V/cm) and 300 mA. The slides were neutralized gently with 0.4 M Tris buffer at pH 7.5 and stained with 75 *μ*L ethidium bromide (20 *μ*g/mL). For positive control, the lymphocytes cells were treated with 100 *μ*M H_2_O_2_ for 10 min at 4°C. Two slides per animal were prepared and 25 cells per slide (250 cells per group) were scored randomly and analyzed using an image analysis system (Komet-5.5, Kinetic Imaging) attached to a fluorescent microscope (Leica) equipped with appropriate filters. The parameter selected for quantification of DNA damage was percent tail DNA as determined by the software.

#### 2.5.8. Histopathology

At the end of the experiment, animals were sacrificed, and fresh portions of the skin from each animals were cut rapidly, fixed in neutral buffered formalin (10%), and then dehydrated using different grades of ethanol (70%, 80%, 90%, 95%, and 100%). Dehydration was followed by clearing the samples in two changes of xylene. The samples were then impregnated with two changes of molten paraffin wax, embedded, and blocked out. The tissue sections (4-5 *μ*m) were stained according to the method described by Bancroft and Stevens [17] using conventional histologic stains. Stained sections from the control and treated mice were observed and photographs were taken using an optical microscope (Olympus, Tokyo, Japan) for alterations in architecture, and for the presence of degeneration, necrosis in skin.

#### 2.5.9. Protein Estimation

The total protein content in skin tissue extracts was estimated by the Bradford method using bovine serum albumin as standard [18].

#### 2.5.10. Statistical Analysis

Design-Expert (Version 8.0) software was used to analyze the experimental data. Values are recorded as mean ± SD. The data obtained from different groups was analyzed by ANOVA. The values *P* < 0.05 were considered statistically significant for all conduct experiments. Every determination was carried out in triplicate.

## 3. Result 

### 3.1. Optimal Condition for Extraction

The effect of extraction temperature, time, and cycles on extraction yield was estimated. According to the above extraction conditions, the yield of *Tinospora cordifolia* was varied in the range from 8.16 to 13.67%. The experimental conditions and the corresponding response values from the experimental design are presented in Table 1. The independent and dependent values were analyzed to obtain a regression equation that could predict the response within the given range. A second-order polynomial equation can be obtained as the follows:

(2) Y=+11.51−0.93X1+3.599E−003X2−0.23X3+1.31X1X2+0.061X1X3+0.2.

**Figure 1:**
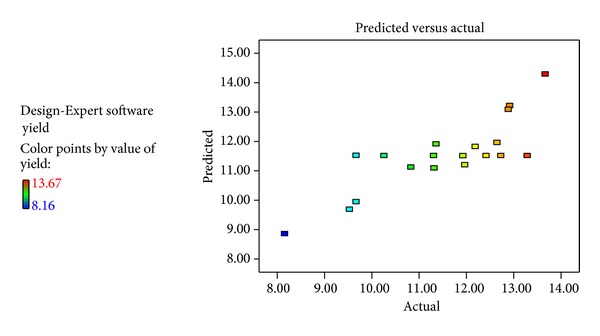
Correlation between predicted and experimental values of the yield of *Tinospora cordifolia.*

**Figure 2:**
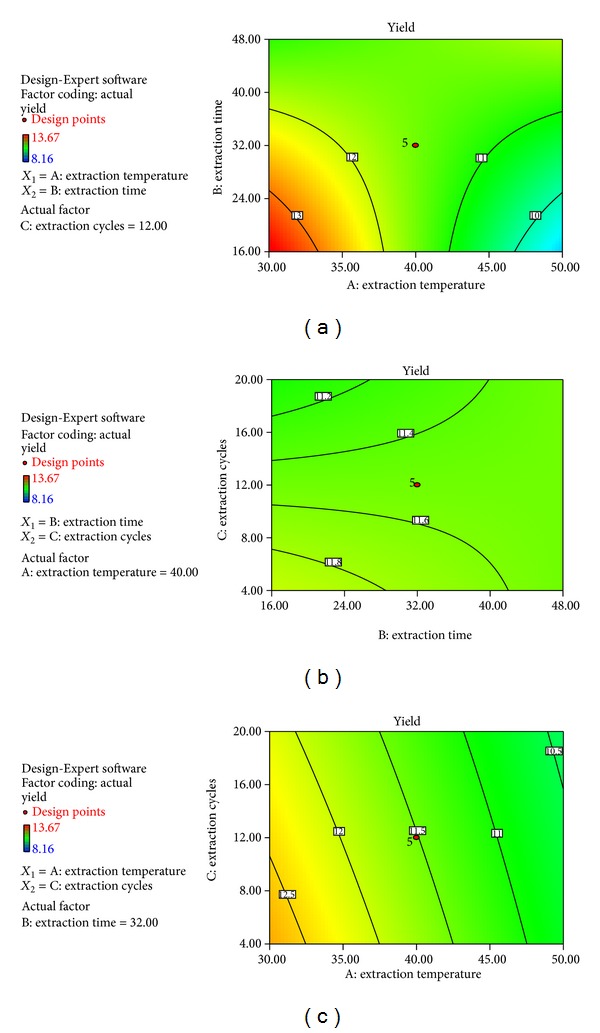
Contour plots showing the effect of extraction temperature, extraction time, and extraction cycle on the yield of *Tinospora cordifolia*.

The plot of the predicted values versus experimental value of the yield of *Tinospora cordifolia* from (2) indicated a good fit, as presented in Figures 1 and 2. Color differences in the fit plot indicated the level of yield which represents red as the highest extracted yield, while narrowed down to blue color was the lowest extracted yield. The developed regression model was adequate and explained the variability for the yield of *Tinospora cordifolia*. The results of analysis of variance gave a coefficient of determination (*R* ^2^) that was 0.666 and adjusted *R* ^2^ that was 0.500 indicating that the developed model would fit well to represent the optimal condition and the relationship among variables. 2FI effects of the factors on the yield of *Tinospora cordifolia* were significant, though the linear, quadratic, and cubic terms were not (see Table 3). An overall model was significant.

The contour plots based on derived equation represent the relationship between the response and the experimental levels of each factor, by which the optimum condition for the maximum yield could be presumed. Different contour plots can reflect the strength of the interaction effects. According to Figure 2, the interaction between the extraction temperature and extraction time was significant (*P* < 0.05). The optimal extraction conditions for the highest yield of *Tinospora cordifolia* were predicted as extraction temperature 40°C, extraction time 16 hours, extraction cycle 4 and the maximum predicted yield was 14.29%.

### 3.2. Effect of Palmatine on DMBA and Croton Oil Induced Skin Carcinogenesis

**Figure 3:**
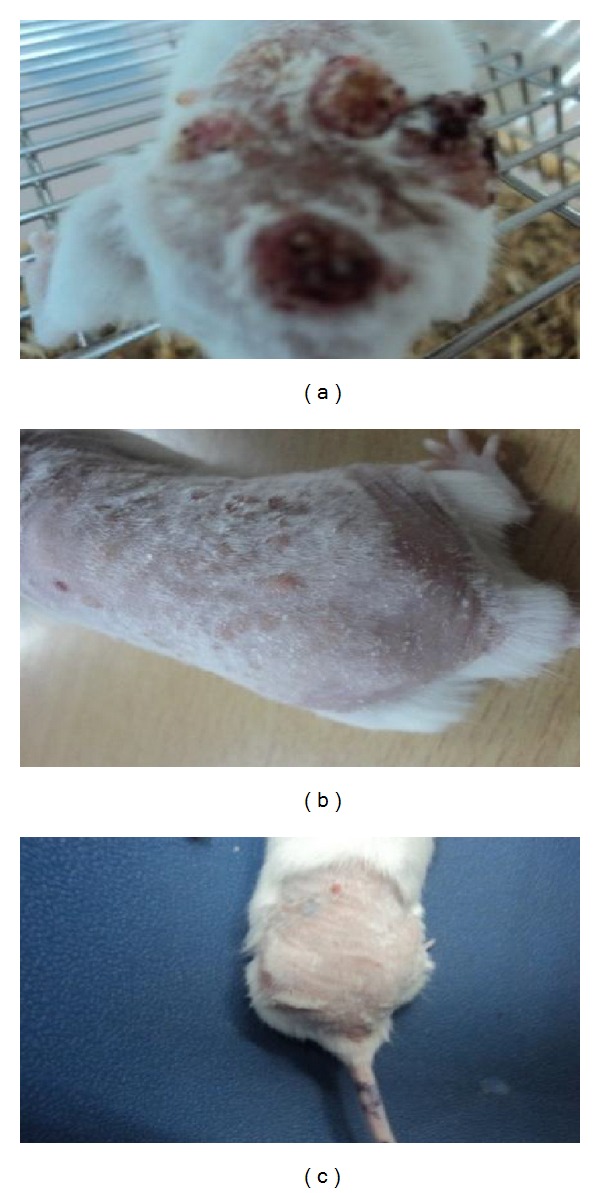
Flavonoid-induced reduction of tumor in Swiss albino mice. (a) Group 2 (water + DMBA + croton oil), (b) Group 4 (palmatine 100 mg/kg + DMBA + croton oil), (c) Group 5 (palmatine 200 mg/kg + DMBA + croton oil).

A gradual decrease in body weight was observed in all animals of the different groups. Animals of Groups 4-5 gave a continuous treatment of palmatine orally as mentioned above along with the repeated application of croton oil and showed a significant reduction in the cumulative number of papillomas and tumor size (Table 4 and Figures 3(b) and 3(c)) as compared to the treated control Group 2 (Figure 3(a)). The latency period was found to be 10.10 ± 5.17 weeks in the carcinogen treated control group, whereas it was significantly increased in palmatine treated groups.

### 3.3. Effect of Palmatine on Enzymes Involved in Oxidative Stress

A significant increase in GSH, SOD, and catalase was noted in the skin of palmatine administered animals (Groups 4 and 5) than in the control group animals (Group 2) (Table 5). On the contrary, the lipid peroxidase level was significantly depleted in palmatine administered animals as compared to control group animals.

### 3.4. Effect of Palmatine on Serum Enzyme Analysis

A significant decrease in enzyme serum glutamate oxalate transaminase and serum glutamate pyruvate transaminase, alkaline phosphatase, and bilirubin level was noted in the serum of palmatine administered animals (Groups 4 and 5) than the control group animals (Group 2) (Table 6).

### 3.5. Histopathology

**Figure 4:**
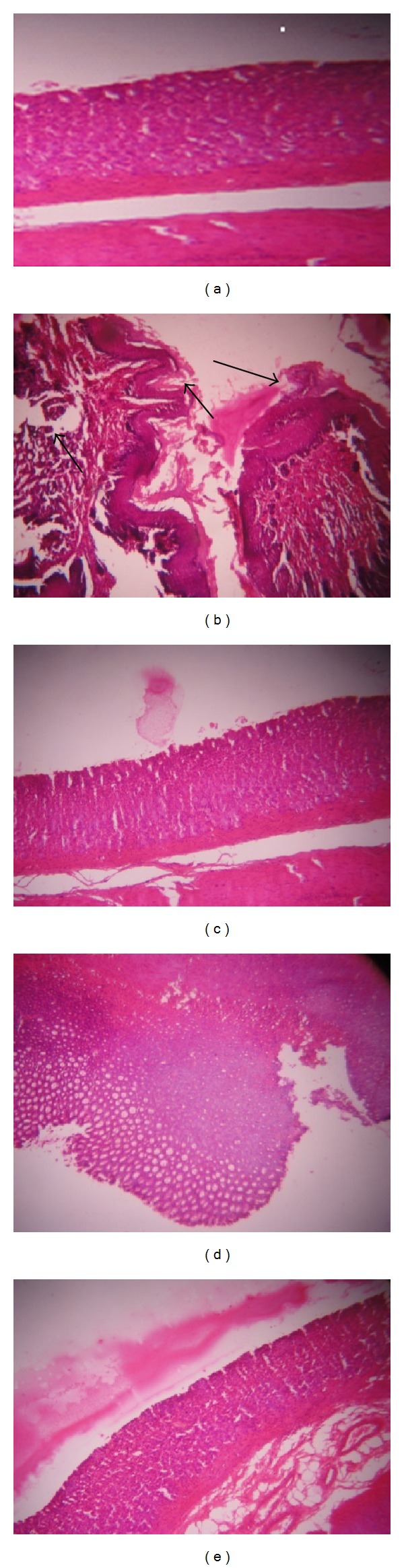
Light microphotographs of cross-sections of mouse skin. (a) Group 1: mouse received normal water demonstrating normal histological architecture,H&E, 400X. (b) Group 2: mouse topical exposure to DMBA + croton oil demonstrating damage ( → ) in dermal layer. H&E. 400X. (c) Group 3-mouse oral and topical exposure to palmatine for 16 weeks showing normal histological architecture. H&E. 400X. (d) Group 4: mouse oral (palmatine 100 mg/kg) and topical (DMBA + croton oil) exposure showing some normal histological architecture, H&E, 400X. Group 5: mouse oral (palmatine 200 mg/kg) and topical (DMBA + croton oil) exposure showing normal histological architecture,H&E, 400X.

In general, the normal dermal layer consists of two layers in which one has loose connective tissue and another has dense connective tissue called the papillary and reticular layers, respectively. It was observed that no alterations occurred in the structure of Group 1 (water treated animal) and Group 3 (only palmatine treated animal) mice skin (Figures 4(a) and 4(c)). on the other hand, DMBA and croton oil treated animals these layers start differentiating in the form of papilloma with signs of abnormal architecture of the epidermal layer and this was due to irregular proliferation of stratum spinosum cells, with abnormal thickening of the stratum corneum and stratum spinosum (Figure 4(b)). Dysplastic changes in the squamous layer and the damage in stroma, hyperkeratosis, acanthosis, and cysts with horns were observed in DMBA and croton oil treated control group (Figure 4(b)). In palmatine treated animals, histological observation (Figures 4(d) and 4(e)) revealed that signs of tumor, hyperkeratosis and acanthosis were present but less than in control Group 2 (Figure 4(b)).

### 3.6. Effect of Palmatine on DNA Damage

**Figure 5:**
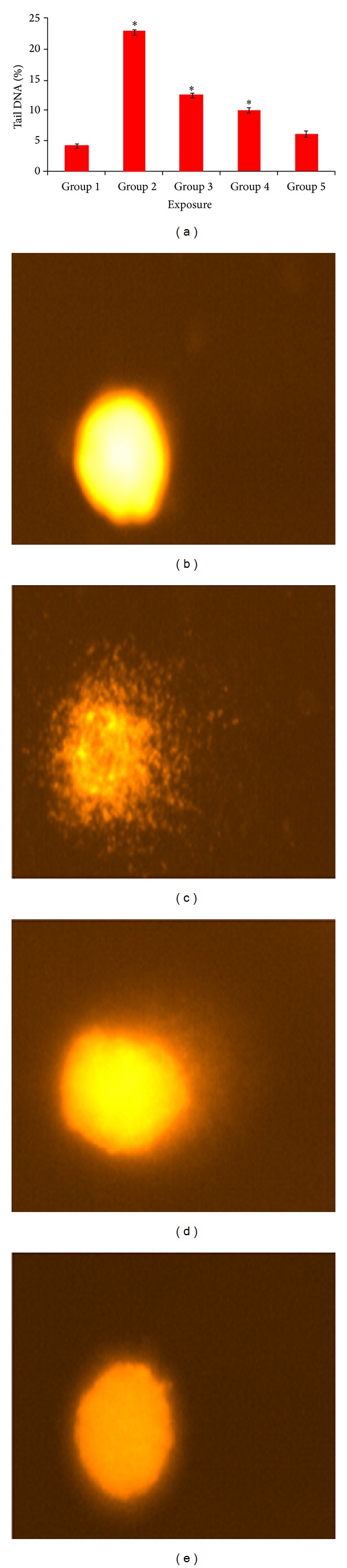
DNA damage in the lymphocytes after different exposures (a) % Tail DNA. (b) Group 1, (c) Group 2, (d) Group 3, and (e) Group 4. Each value represents the mean ± S.E. of three experiments. *P < 0.05 versus control.

The DNA damage was measured as % tail DNA in the control (Groups 1-2) as well as exposed Groups 3–5. The animal exposed to DMBA and croton oil exhibited significantly (*P* > 0.05) higher DNA damage in lymphocytes cells than those of control and Groups 3–5 (Figure 5).

## 4. Discussion

The suitability of the model equation for predicting the optimum response values was evaluated using the optimal conditions. When the contour plots are oval, it means that the interaction of two independent variables is significant. In contrast, the round contour plots are considered not significant [19]. In the present study, the interaction between extraction time and temperature was significant. A model is considered an adequate one if the predicted values are observed during the validation test [20]. Here, the observed values are very close to the predicted values. So, optimum conditions for extraction method were verified.

Human epidemiological data indicate that regular use of certain medicinal plants suppresses carcinogenesis in various organs [21]. So, it is becoming increasingly important to screen natural plant products which might suppress or reverse the process of carcinogenesis [22]. The results of the current study, and many others, indicate several beneficial effects of alkaloids. Skin carcinogenesis is based on the fact that initiation with the sequential application of a subthreshold dose of a carcinogen such as DMBA, followed by repetitive treatment with a noncarcinogenic promoter-like croton oil will lead to the development of skin tumors. Among the initiation and promotion steps, animal studies show that the promotion stage takes longer period to occur and it is reversible initially [23]. Therefore, cancer prevention by inhibition of tumor promotion is expected to be a resourceful approach. In the present study, palmatine administration could extensively inhibit DMBA induced papilloma formation both in terms of incidence of tumor as well as the mean number of papillomas.

Lipid peroxidation is a free radical chain reaction and is known to cause two main steps of carcinogenesis, that is, initiation and propagation. It is a highly destructive process. During the carcinogenic process, lipid peroxidation is increased and more complex and reactive compounds such as malondialdehyde (MDA) and 4-hydroxynonenal were formed. These products of lipid peroxidation were observed to be mutagenic and carcinogenic [24]. It is therefore implied that agents that can reduce the production of free radicals *in vivo* may be considered to have the potential for chemoprevention [25]. In the present study, administration of palmatine significantly reduced the level of LPO in mice exposed to DMBA and croton oil and subsequently decreases the incidence of skin tumor.

Glutathione (GSH) is a tripeptide nonenzymatic antioxidant with a single cysteine residue and constitutes an important pathway of the antioxidant and detoxification defense. GSH is essential for protection of the cells against reactive oxygen species and free radicals produced even in normal metabolism [26]. Due to its polyfunctional properties, GSH plays an important role in drug metabolism, radiation and cancer, immunology, aging, and exercise [27–29]. In the present study, palmatine alkaloid offered protection against carcinogenicity that can be attributed to an elevation in the glutathione level that could have been mediated through the modulation of cellular antioxidant level. In the control group, the activities of GSH were decreased, while palmatine treatment increased the GSH level, which clearly suggests their antioxidant property. Antioxidants are reported to possess a chemopreventive property. Antioxidants are generally regarded as the first line of defense against free radical stress and suggest their usefulness in eliminating the risk of oxidative damage induced during carcinogenesis. SOD and catalase are acting as mutually supportive antioxidative enzymes, which provide protective defense against reactive oxygen species [30]. There was a significant enhancement in the level of GSH, SOD, and catalases in the palmatine treated group compared to control group animals.

DMBA treatments induce lipid peroxidation and reactive oxygen species in the affected area of the skin and ultimately lead to carcinogenesis. This oxidative stress was easily observed in the control group as level of LPO was higher and the level of catalase, SOD, and GSH was lower. The beneficial action of palmatine is probably due to its ability to stimulate the antioxidant enzymes in the cells. This increase in enzyme activity effectively reduced the generation of ROS and LPO in the skin and thus might reduce the incidences of skin papillomas on the treated areas has reported that extensive DNA damage triggers apoptosis [31]. However, some researchers have reported that strand breaks may be introduced directly by carcinogenic compounds by induction of apoptosis or necrosis, indirectly through interaction with oxygen radicals or other reactive intermediates, or as a consequence of excision repair enzymes [32]. DMBA and croton oil treated group showed highly fragmented nuclei showing damage to the head region and images with nearly all DNA in the tail or with a very wide tail were observed more frequently. Superoxide dismutase is specialized to convert the highly toxic superoxide radical to less toxic H_2_O_2_. The catalase enzyme reduces H_2_O_2_ to H_2_O. We measured blood biochemical parameters, including enzymes, to evaluate organ function in our experimental animals. There was a significant decrease in GOT, GPT, ALP, and bilirubin levels as compared to Group 2 (Table 6). The recovery of histologic changes observed in the skin and the accompanying decrease in ALP and GOT levels after coexposure of palmatine with DMBA and croton oil treated group. Reactive oxygen species typically include the superoxide radical (O_2_^−^), H_2_O_2_, and the hydroxyl radical (OH^•^), which cause damage to cellular components, including DNA, and ultimately lead to apoptotic cell death.

## 5. Conclusion

In conclusion, our results indicate that the extraction condition for *Tinospora cordifolia *were optimized by central composite design and 2FI polynomial model was obtained from RSM. The optimal yield of* Tinospora cordifolia *which was obtained from experiments closely matched the predicted yield. Palmatine isolated from *Tinospora cordifolia* could notably enhance the antioxidant enzyme levels for antioxidant enzyme activity like GSH, SOD, catalase, and inhibited lipid peroxidation hence showing its role in detoxification pathway. Both enzyme activities and histological analysis suggest that environmental carcinogens that induce skin carcinogenesis can be inhibited by oral administration of palmatine in the daily diet to achieve some protection against skin cancer. The results from the present study indicate that different samples of palmatine can inhibit papilloma growth. 

## Figures and Tables

**Table 1:** Independent variables and their levels used in the response surface design.

Serial number	Independent variables	Symbols	Factor level		
			Low (−1)	Middle (0)	High (+1)
1	Extraction temperature (°C)	*X*_1_	30	40	50
2	Extraction time (hours)	*X*_2_	16	32	48
3	Extraction cycles (cycle)	*X*_3_	4	12	20

**Table 2:** Central composite design by RSM program for optimization of extraction conditions with experimental and their corresponding predicted yields.

Runs	Extraction temperature (*X*_1_, °C)	Extraction time (*X*_2_, hours)	Extraction cycles (*X*_3_, cycle)	Experimental yield (%)	Predicted yield (%)
1	40 (0)	32 (0)	12 (0)	13.29	11.51
2	50 (1)	16 (−1)	20 (1)	8.16	8.86
3	40 (0)	32 (0)	12 (0)	10.26	11.51
4	30 (−1)	48 (1)	20 (1)	11.32	11.09
5	23.18 (−1.68)	32 (0)	12 (0)	12.89	13.09
6	56.82 (1.68)	32 (0)	12 (0)	9.67	9.94
7	40 (0)	5.09 (−1.68)	12 (0)	11.93	11.51
8	40 (0)	32 (0)	−1.45 (−1.68)	11.36	11.91
9	50 (1)	48 (1)	4 (−1)	12.19	11.82
10	40 (0)	32 (0)	25.45 (1.68)	10.83	11.12
11	30 (−1)	48 (1)	4 (−1)	11.97	11.20
12	40 (0)	32 (0)	12 (0)	11.31	11.51
13	30 (−1)	16 (−1)	04 (−1)	13.67	14.29
14	50 (1)	16 (−1)	04 (−1)	9.53	9.68
15	40 (0)	58.91 (1.68)	12 (0)	9.67	11.52
16	40 (0)	32 (0)	12 (0)	12.73	11.51
17	50 (1)	48 (1)	20 (1)	12.65	11.96
18	40 (0)	32 (0)	12 (0)	12.42	11.51
19	30 (−1)	16 (−1)	20 (1)	12.92	13.21

**Table 3:** Analysis of variance for fitted 2FI polynomial model.

Source	Sum of square	Degree of freedom	Mean square	*F*-value	*P* value
Model	26.83	6	4.47	4.00	0.019 significant
Residual	13.40	12	1.12		
Lack of fit	7.52	8	0.94	0.64	0.726 not significant
Pure error	5.88	4	1.47		
Cor total	40.23	18			

**Table 4:** Chemopreventive effect of palmatine against DMBA and croton oil induced skin carcinogenesis in mice.

Treatment	Final body weight (g)	Number of papilloma	Tumor size	Average latency period^a^
Group 1	37.56 ± 5.43*			
Group 2	22.75 ± 11.24	10.16 ± 5.03	2.06 ± 0.37	10.10 ± 5.17
Group 3	36.96 ± 6.31*			
Group 4	38.87 ± 9.67*	1.50 ± 1.04*	0.90 ± 0.29*	15.73 ± 0.58*
Group 5	40.12 ± 6.89*	0.83 ± 0.75*	0.56 ± 0.22*	18.16 ± 0.55*

Values with * superscripts were significant (*P* < 0.05) in comparison with Group 2.

^a^The lag between the application of the promoting agent and the appearance of 50% of tumors was determined. Average latency period = ∑*fx*/*n*; *f* is the number of tumors appearing each week; *x* is the numbers of weeks; and *n* is the total number of tumors.

**Table 5:** Inhibition of dimethylbenz(a)anthracene (DMBA)/croton oil induced skin carcinogenesis in Swiss albino mice by palmatine treatment.

Treatment	GSH *μ*mole/mg protein	SOD *μ*mole/mg protein	Catalase U/mg protein	LPO nmole/mg protein
Group 1	24.28 ± 1.60*	74.42 ± 2.06*	36.60 ± 1.72*	1.82 ± 0.86*
Group 2	11.02 ± 0.96	59.59 ± 2.15	8.97 ± 1.07	4.82 ± 1.71
Group 3	26.68 ± 1.20*	73.10 ± 1.56*	37.50 ± 2.40*	2.10 ± 0.76*
Group 4	28.05 ± 2.83*	77.50 ± 0.84*	38.84 ± 3.18*	2.29 ± 0.80*
Group 5	32.09 ± 0.90*	84.05 ± 3.78*	41.61 ± 3.61*	1.94 ± 0.49*

Values with * superscripts were significant (*P* < 0.05) in comparison with Group 2.

**Table 6:** The effect of palmatine on serum enzyme and bilirubin levels in mice.

Treatment	SGOT	SGPT	SALP	Bilirubin mg/dL
	IU/L	IU/L	IU/L	
Group 1	58.33 ± 1.47*	34.45 ± 1.75*	30.29 ± 0.73*	1.12 ± 0.03*
Group 2	162.73 ± 12.33	149.06 ± 17.00	231.52 ± 13.93	5.56 ± 2.53
Group 3	56.48 ± 2.65*	31.46 ± 1.25*	42.89 ± 3.07*	0.97 ± 0.12*
Group 4	101.24 ± 9.86*	63.75 ± 24.49*	68.85 ± 7.05*	2.17 ± 1.65*
Group 5	80.91 ± 12.17*	49.02 ± 24.06*	55.76 ± 3.53*	1.95 ± 0.76*

Values with * superscripts were significant (*P* < 0.05) in comparison with Group 2.
